# Birdlike growth and mixed-age flocks in avimimids (Theropoda, Oviraptorosauria)

**DOI:** 10.1038/s41598-019-55038-5

**Published:** 2019-12-11

**Authors:** G. F. Funston, P. J. Currie, M. J. Ryan, Z.-M. Dong

**Affiliations:** 1 Department of Biological Sciences, University of Alberta, Edmonton, AB Canada; 2 Department of Earth Sciences, Carleton University, Ottawa, ON Canada; 3 Institute of Vertebrate Paleontology and Paleoanthropology, Beijing, China

**Keywords:** Palaeontology, Animal behaviour

## Abstract

Avimimids were unusual, birdlike oviraptorosaurs from the Late Cretaceous of Asia. Initially enigmatic, new information has ameliorated the understanding of their anatomy, phylogenetic position, and behaviour. A monodominant bonebed from the Nemegt Formation of Mongolia showed that some avimimids were gregarious, but the site is unusual in the apparent absence of juveniles. Here, a second monodominant avimimid bonebed is described from the Iren Dabasu Formation of northern China. Elements recovered include numerous vertebrae and portions of the forelimbs and hindlimbs, representing a minimum of six individuals. Histological sampling of two tibiotarsi from the bonebed reveals rapid growth early in ontogeny followed by unexpectedly early onset of fusion and limited subsequent growth. This indicates that avimimids grew rapidly to adult size, like most extant birds but contrasting other small theropod dinosaurs. The combination of adults and juveniles in the Iren Dabasu bonebed assemblage provides evidence of mixed-age flocking in avimimids and the onset of fusion in young individuals suggests that some of the individuals in the Nemegt Formation bonebed may have been juveniles. Regardless, these individuals were likely functionally analogous to adults, and this probably facilitated mixed-age flocking by reducing ontogenetic niche shift in avimimids.

## Introduction

**Figure 1:**
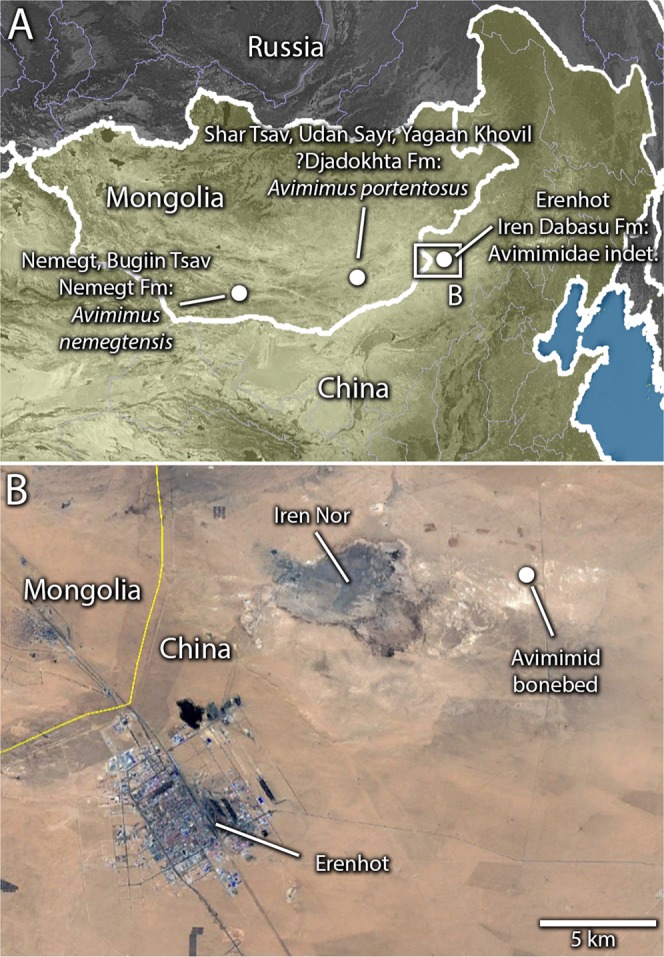
Geographical setting of the Iren Dabasu avimimid bonebed. Map of Eastern Asia (**A**) showing localities where avimimids have been found in China and Mongolia (highlighted; inset shows location of image **B**). Satellite image (**B**) of Erenhot Region, showing Iren Nor and the location of the Iren Dabasu avimimid bonebed. Map data and satellite images in (**A**,**B**) from Google Maps (map data: © Google Maps), used under fair use terms.

Avimimidae was an enigmatic, monogeneric family of oviraptorosaurs from China and Mongolia (Fig. 1). *Avimimus* was first described by Kurzanov^1^ and its bird-like morphology immediately confused palaeontologists. Although regarded as a non-avian theropod by Kurzanov^1^, other workers interpreted its mosaic of features as similar to those of a flightless avian^2^, a sauropod^3^, and even an ornithopod dinosaur^3^. These apparently contradictory hypotheses led several authors^4,5^ to suggest that the holotype may have been a chimaera, a possibility Kurzanov considered himself^4^. However, Vickers-Rich *et al*.^5^ argued this claim, and the subsequent discovery of an articulated skeleton^6^ indicated that the material did indeed belong to a single taxon. The oviraptorosaurian affinities of *Avimimus* were first recognized by Maryanska *et al*.^7^, although their analysis also placed oviraptorosaurs within Avialae, a conclusion no longer supported by broad-scale theropod phylogenies^8,9^. Recent analyses^10–12^ have recovered *Avimimus* as an intermediate oviraptorosaur, sister to Caenagnathoidea (=Caenagnathidae + Oviraptoridae).

The rarity of avimimid material and its poor history of description has led to several problems in the understanding of these animals. A series of detailed descriptions of *Avimimus portentosus* in Russian by Kurzanov^13–17^, and their translations, comprise most of the literature on avimimids. Despite the discovery of numerous additional specimens in the intervening years, few of these have been described until recently. An expedition led by the Hayashibara Museum of Japan collected a nearly complete skeleton including cranial material (MPC-D 100/129) from Shar Tsav in the eastern Gobi Desert of Mongolia, but it has not been described beyond conference abstracts^6^. In 2006, the same organization discovered a second skeleton in the Nemegt Formation at Bugiin Tsav, in western Mongolia, the cranium of which was recently described^18^. A bonebed of disarticulated avimimids from the Nemegt Formation of Mongolia was discovered in 2006^19^, but was not described until ten years later^20^. Subsequent examination of that material determined that it represents a new species, *Avimimus nemegtensis*, based on a suite of cranial and postcranial differences from the holotype of *Avimimus portentosus*^21^.

A second bonebed from the Iren Dabasu Formation of China was briefly described by Ryan *et al*.^22^, but has not received further attention until now. The general age of the Iren Dabasu Formation is widely accepted as Late Cretaceous, but its precise age is debated. Granger and Berkey^23^ indicated a Cretaceous age, but did not speculate further. Morris^24^ hypothesized that the beds were Campanian, but others since have suggested an older age on the basis of a relatively primitive dinosaur fauna^25,26^. Currie and Eberth^27^ reevaluated the biostratigraphy and sedimentology of the Iren Dabasu Formation and drew similarities with the Bayn Shiree Formation exposed at Bayshin Tsav. They concluded that these sediments were probably early Senonian (=Cenomanian–Santonian), but that several unusual members of the fauna (avimimids and troodontids) may point to a Campanian age. Van Itterbeeck *et al*.^28^ described microfossil data supporting a Campanian–Maastrichtian age for the formation and suggested it might correlate to the Nemegt Formation further west. Averianov and Sues^29^ used vertebrate biostratigraphy and found evidence for a Santonian age, arguing that the microfossil similarity of the Nemegt and Iren Dabasu Formations was the result of similarity in environment rather than age. Most recently, Bonnetti *et al*.^30^ used palynostratigraphy to support the assertion of Van Itterbeeck *et al*.^28^ that the Iren Dabasu (=Erlian) Formation is late Campanian–Maastrichtian. Regardless of the equivocal age of the Iren Dabasu Formation, its paleoenvironment is well established. The sedimentology indicates a terrestrial fluvial system with braided channels in a semi-arid climate^27^. Preservation of egg nests, caliche and paleosols indicates periodic subaerial exposure, and the presence of plesiosaurs and hybodont sharks indicates a river system with a marine connection.

**Figure 2:**
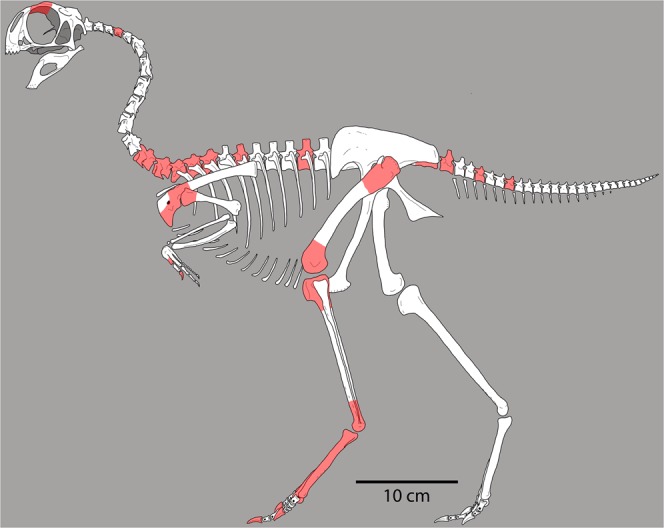
Skeletal reconstruction of a generalized avimimid, showing elements recovered at the Iren Dabasu bonebed (shaded). Reconstruction based on *Avimimus nemegtensis* and *Avimimus portentosus* (MPC-D 100/129). For clarity, known appendicular elements are shaded on the left side only, regardless of which side(s) are represented in the bonebed.

The avimimid bonebed site described herein was originally discovered by a Sino-Soviet expedition in 1959, which used bulldozers to excavate the site. It was revisited in 1987 and 1988 by the Sino-Canadian expedition and numerous fragmentary bones representing all regions of the skeleton (Fig. 2) were recovered from the spoil piles left by the Sino-Soviet bulldozers. Unfortunately, the material collected by the Sino-Soviet expedition still awaits preparation and it may never be available for study. Here we describe the bonebed assemblage collected by the Sino-Canadian expedition and perform histological analyses to provide further insight on the growth of avimimids and the process of tibiotarsal fusion in these animals.

## Institutional Abbreviations

AMNH, American Museum of Natural History, New York, New York, USA; IVPP, Institute of Vertebrate Paleontology and Paleoanthropology, Beijing, China; MPC, Institute of Paleontology and Geology, Mongolian Academy of Sciences, Ulaanbaatar, Mongolia; UALVP, University of Alberta Laboratory for Vertebrate Paleontology, Edmonton, Alberta, Canada.

## Results

### Systematic paleontology

Dinosauria Owen, 1842^31^

Saurischia Seeley, 1888^32^

Theropoda Marsh, 1881^33^

Coelurosauria Huene, 1914^34^

Maniraptora Gauthier, 1986^35^

Oviraptorosauria Barsbold, 1976^36^

Avimimidae Kurzanov, 1981^1^

Gen. et sp. indet.

### Referred specimens

IVPP V16313–14, V16316–19, V16321–45, disarticulated bonebed float comprising vertebrae, forelimb, and hindlimb elements (Fig. 2).

### Horizon and locality

Iren Dabasu Fm. (?Campanian), Erenhot (Iren Dabasu), Nei Mongol, China.

### Description

#### Axial skeleton

**Figure 3:**
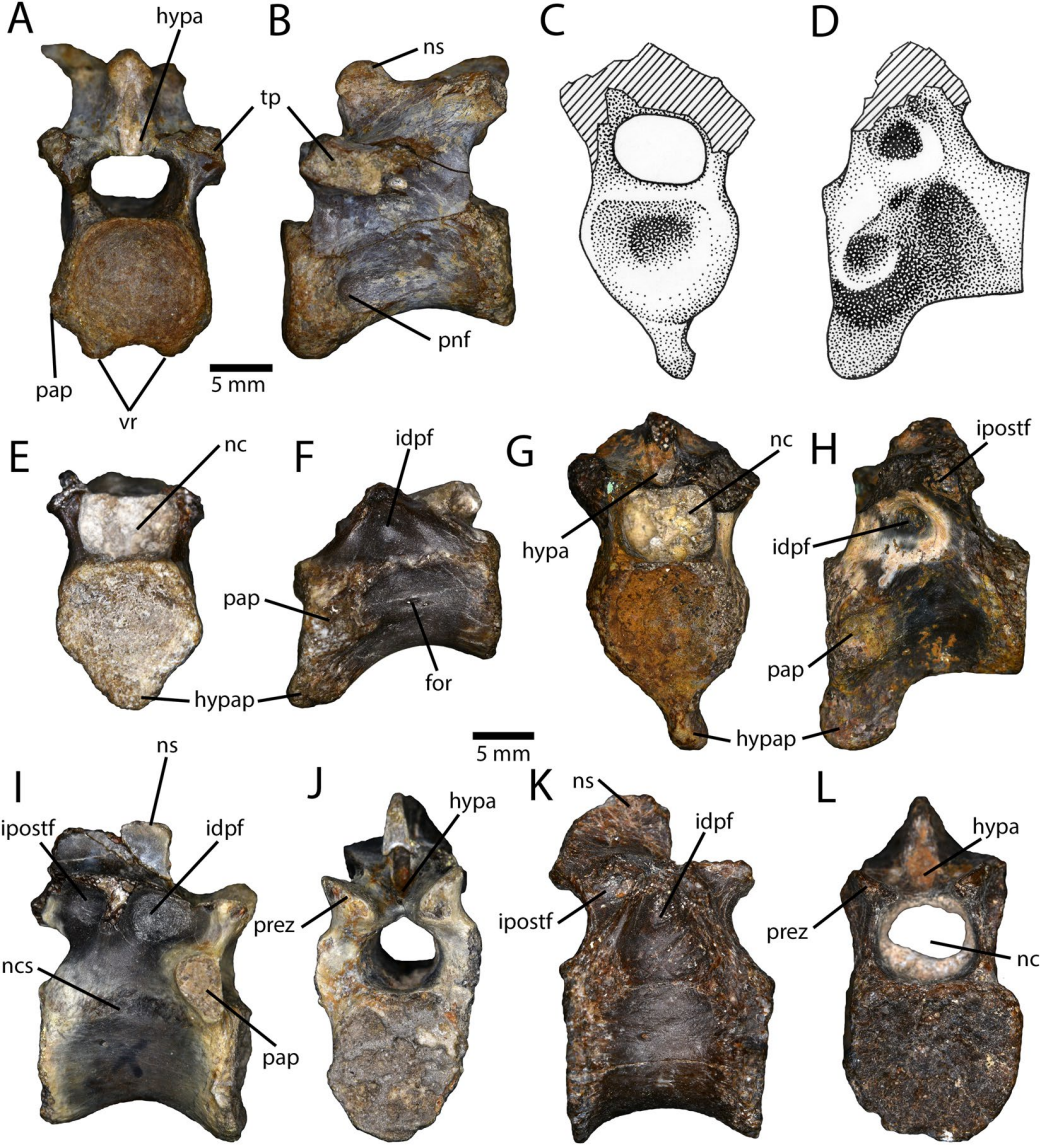
Presacral vertebrae from the Iren Dabasu avimimid bonebed. Posterior cervical vertebra (IVPP V16329.a) in anterior (**A**) and dorsal (**B**) views. Illustrations (**C**,**D**) and photographs (**G**,**H**) of second cervicodorsal (IVPP V16332.a) in anterior (**C**,**G**) and lateral (**D**,**H**) views. First cervicodorsal vertebra (IVPP V16332.b) in anterior (**E**) and lateral (**F**) views. Anterior dorsal (IVPP V16318.a) in lateral (**I**) and anterior (**J**) views. Posterior dorsal (IVPP V16318.b) in lateral (**K**) and anterior (**L**) views. Abbreviations: for, foramen; hypa, hypantrum; hypap, hypapophysis; idpf, infradiapophyseal fossa; ipostf, infrapostzygapophyseal fossa; nc, neural canal; ncs, neurocentral suture; ns, neural spine; pap, parapophysis; pnf, pneumatic fossa; prez, prezygapophysis; tp, transverse process; vr, ventral ridges.

**Figure 4:**
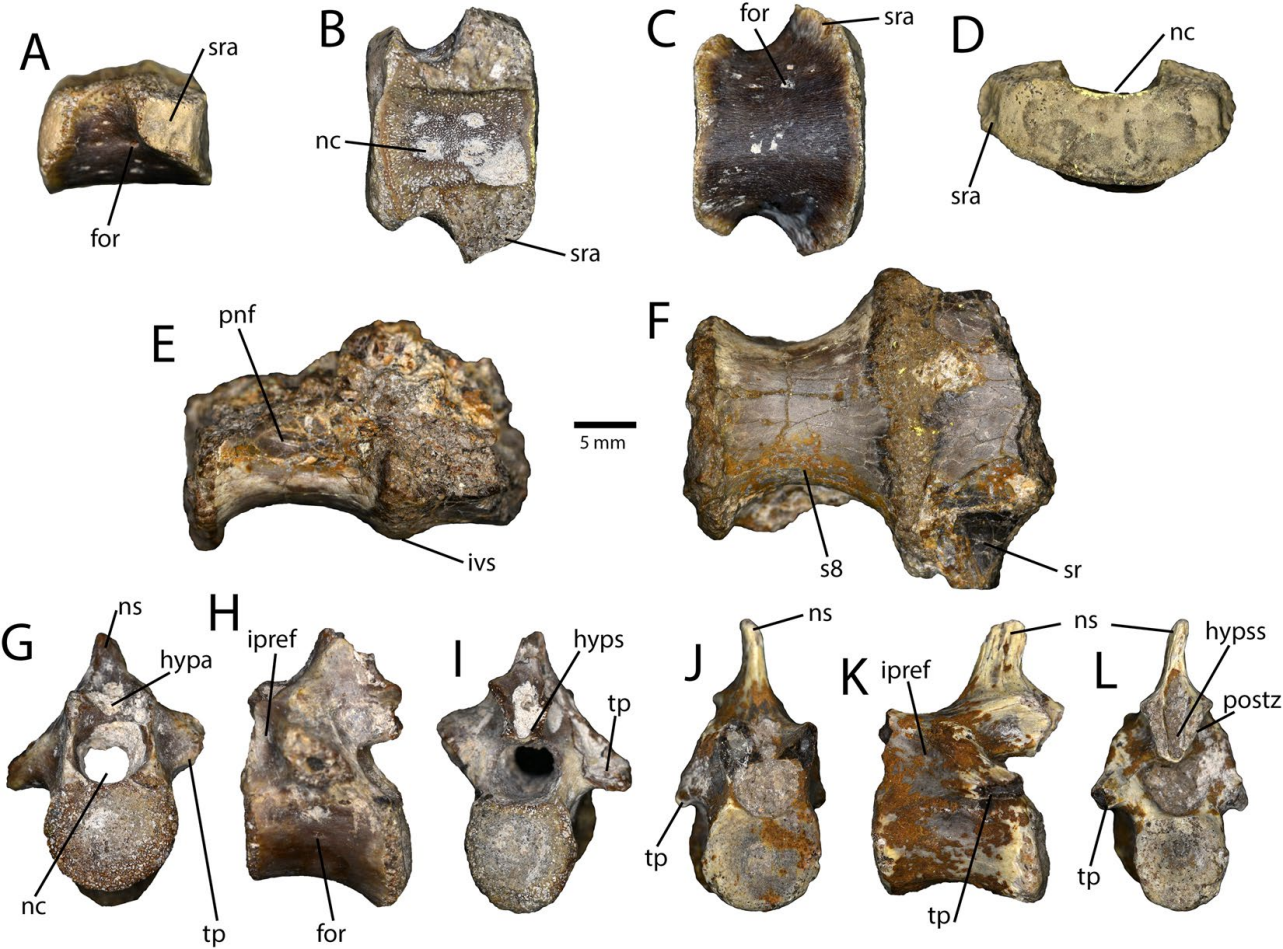
Sacral and caudal vertebrae from the Iren Dabasu avimimid bonebed. Isolated juvenile mid-sacral vertebra (IVPP V16328) in lateral (**A**), dorsal (**B**), ventral (**C**), and anterior (**D**) views. Partial sacrum (IVPP V16330) in lateral (**E**) and ventral (**F**) views. Proximal caudal vertebra (IVPP V16317.a) in anterior (**G**), lateral (**H**), and posterior (**I**) views. Mid-caudal vertebra (IVPP V16317.b) in anterior (**J**), lateral (**K**), and posterior (**L**) views. Abbreviations: for, foramen; hypa, hypantrum; hyps, hyposphene; hypss, hyposphenal slot; ipref, infraprezygapophyseal fossa; ivs, intravertebral suture; nc, neural canal; ns, neural spine; pnf, pneumatic fossa; postz, postzygapophysis; s8, sacral vertebra eight; sr, sacral rib; sra, sacral rib attachment; tp, transverse process.

An assortment of vertebrae from all regions of the spinal column (Figs. 3 and 4) were recovered, including three cervicals, four cervicodorsals, six dorsals, three partial sacra, and nine caudals. Some other vertebrae were also collected, but they differ from those of avimimids and likely represent another taxon.

The smallest cervical vertebra (IVPP V16318) is likely from a skeletally immature individual because the neurocentral sutures are open. It has vertical articulating surfaces, which suggests it is from the posterior half of the neck, based on comparison to MPC-D 100/129 and PIN 3907/1^5^ (*Avimimus portentosus*). There is a small pleurocoel on each side and numerous smaller foramina pierce the centrum. IVPP V16329.a (Fig. 3A,B) is the most complete and probably represents the last or second last cervical. The articular faces are vertical and shallowly concave. There are two prominent ventral ridges on the centrum, although they do not extend far posteriorly. Lateral to these ridges is a prominent parapophysis and posterior to this is a deep fossa in place of a pleurocoel (Fig. 3B). The neurocentral suture is completely obliterated and the neural arch lacks pneumatic excavation except for two small post-diapophyseal fossae. The neural spine is short and square and there is a small anterolateral knob on each side. The hypantral facets are well developed and there is a deep slot dorsal to the hyposphene. The epipophyses are very small compared to those of other oviraptorosaurs, but this could be the result of allometry.

Material from the bonebed suggests that this avimimid had three cervicodorsal vertebrae, whereas *Avimimus portentosus* (MPC-D 100/129 and PIN 3907/1^5^) apparently has only two. Two of the cervicodorsal vertebrae (IVPP V16332.a and IVPP V16329.b) appear to be equivalent to the first cervicodorsal vertebra of *Avimimus portentosus* (MPC-D 100/129). However, another vertebra (IVPP V16332.b; Fig. 3) seems more similar to the last cervical vertebra, but lacks a pleurocoel and has a prominent hypapophysis. Accordingly, it is better categorized as a first cervicodorsal, rather than cervical, vertebra. Therefore, the first cervicodorsal vertebra of *Avimimus portentosus* is equivalent to the second cervicodorsal vertebra of this animal. The first cervicodorsal vertebra (IVPP V16332.b; Fig. 3A,B) has a rounded, bulbous, anteriorly projecting hypapophysis. Dorsolateral to the hypapophysis, there are rounded parapophyses with small foramina posterior to them. The articular face is moderately concave. The neurocentral suture is fused but still open. There are incipient infradiapophyseal fossae on the neural arch. One of the second cervicodorsal vertebrae (IVPP V16329.b) has a broken hypapophysis, whereas in the other (IVPP V16332.a; Fig. 3C,D) it is tab-like and prominent, with a rounded ventral edge. It projects slightly anteroventrally, but asymmetrically so, being directed more to the left side of the animal than the right (Fig. 3C), which does not appear to be the result of post-burial deformation. The parapophyses are concave and sit on prominent laterally projecting mounds of bone. They vary in shape, being either circular or kidney-shaped. The anterior articular surface is slightly concave and the posterior articular surface is flat. There are no foramina in the centrum and the neurocentral sutures are closed in both specimens. Both specimens have infradiapophyseal and infrapostzygapophyseal fossae, but they are relatively deeper in IVPP V16332.a. In IVPP V16329.b, an extra fossa is situated on the ventrolateral surface of the postzygapophysis, and it is discontinuous with the infrapostzygapophyseal fossa. In both specimens, the neural spine is small, there is a deep slot posterior to it above the hyposphene, and there is an anteriorly facing pit between the hypantra. A third cervicodorsal vertebra (IVPP V16323.a) can be identified based on the presence of a small hypapophysis and large parapophyses on the centrum. It lacks most of the neural arch, but the neurocentral suture is closed.

Six dorsal vertebrae (Fig. 3C–L) were collected and, although it is likely that they represent different regions of the back, none can be confidently identified to a specific position. All of the dorsal vertebrae lack lateral pleurocoels, and all but one have closed neurocentral sutures. Three of the vertebrae have parapophyses preserved, and in each case they are elongate and tear-drop shaped, extending across the neurocentral suture. These three vertebrae also have ventral keels, which indicates that they are from the anterior part of the dorsal column. One vertebra (IVPP V16318.b) has a complete neural arch, but lacks parapophyses (Fig. 3K,L), indicating it is from the posterior part of the series. The neural arches of the remaining vertebrae are broken, but no parapophyses are present on the centra of these vertebrae, so they are likely from the middle or posterior part of the series. The dorsal vertebrae have infradiapophyseal and infrapostzygapophyseal fossae, the latter of which are deeper (Fig. 3I). The neural spine is low, anteroposteriorly long, and square in lateral view. There is a deep slot anterior to the neural spine for the hypantrum-hyposphene contact, and a deep slot above the hyposphene.

Three partial sacra were recovered. One of these consists of a single, unfused sacral vertebra (IVPP V16328; Fig. 4A–D), whereas the other specimen consists of two fused vertebrae. The latter specimen (IVPP V16330; Fig. 4E,F) is from the posterior end of the sacrum, based on the height and width of the centra and the positions of the sacral ribs. Of these, IVPP V16330 is both larger and more complete, but also more poorly preserved. The centra lack ventral ridges or grooves, and none have lateral pleurocoels. The neurocentral sutures of the smaller specimen appear unfused and, as a result, the neural arch is not preserved (Fig. 4B). The neural arch of the larger specimen is preserved, but badly damaged, and the neurocentral suture is not visible. The isolated sacral vertebra (IVPP V16328) is from somewhere in the middle of the sacrum, based on the laterally deflected facets for the sacral ribs (Fig. 4A–D). The neural arch is missing and there is a clean neurocentral suture, indicating that the neural arch had not yet fused. Similarly, the articular surfaces of the centrum are complete, which indicates that this vertebra had not yet fused to the others in the sacrum. Combined with porous, striated bone texture, this suggests that this individual was young at the time of death. Numerous small foramina pierce the lateral sides of the centrum, but these probably reflect the young age of the individual rather than pneumatization of the vertebra.

The caudal vertebrae range in size and likely position, although none can be identified to an exact position. The more proximal caudal vertebrae (Fig. 4H) have a distinct disparity in the locations of the articular faces: the posterior face is positioned further ventrally when both faces are oriented vertically. On some of the vertebrae, this disparity is associated with a flat or grooved ventral surface of the centrum, whereas those with less disparity tend to be ventrally rounded. Invariably, the caudal vertebrae lack lateral pleurocoels and any pneumatic fossae on the neural arches. Where preserved, the transverse processes sweep posteriorly (Fig. 4K), which tends to be the case in oviraptorosaurs, except for the distal caudal vertebrae, where they are oriented transversely or anteriorly. The prezygapophyses are long and face medially. The postzygapophyses are short and the slot between them is reduced compared to the deep supra-hyposphenal slots of the dorsal vertebrae (Fig. 4I,L). The neural spine is relatively tall and located above the posterior half of the vertebra.

#### Appendicular skeleton

**Figure 5:**
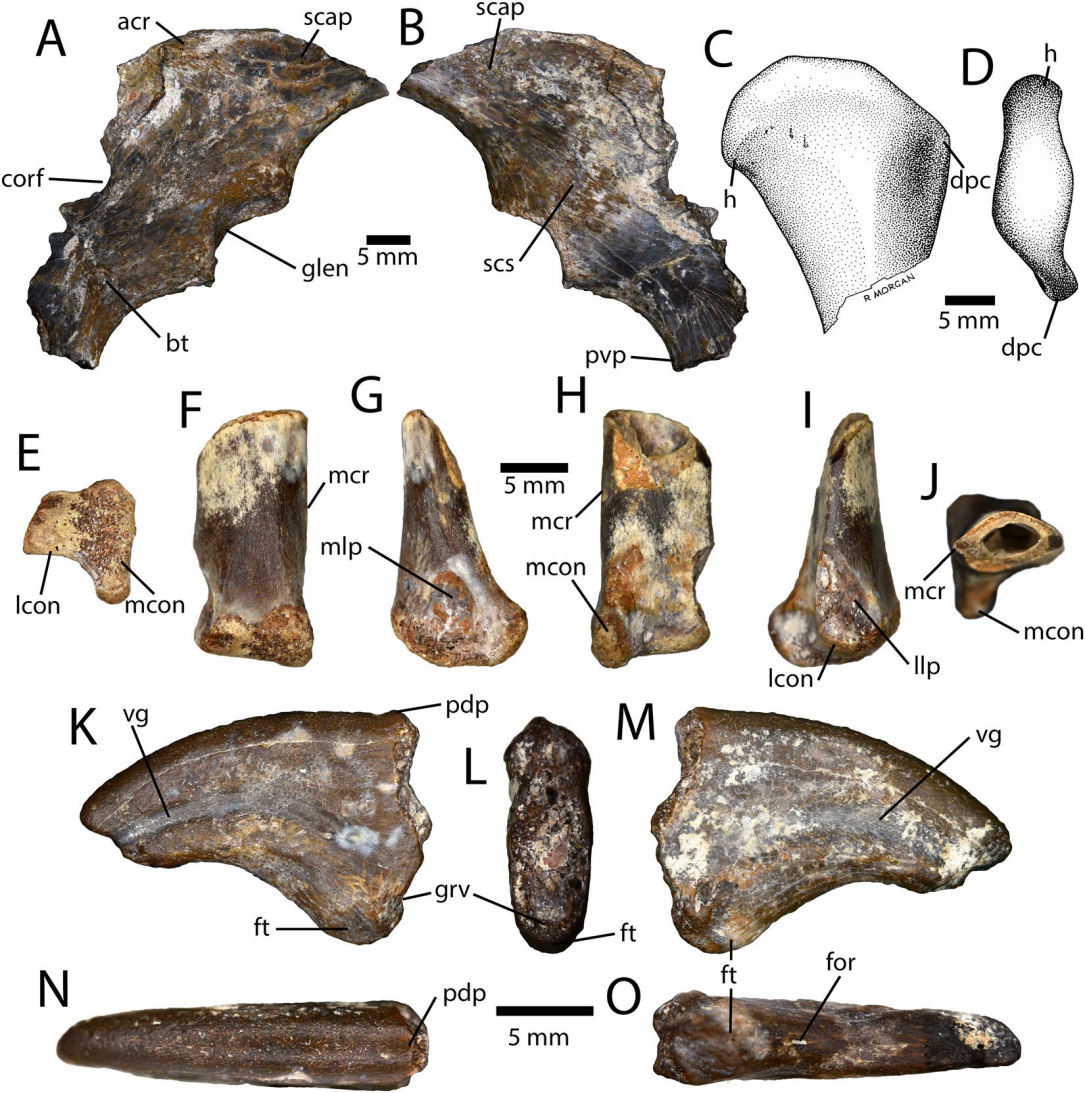
Forelimb material from the Iren Dabasu avimimid bonebed. Partial scapulacoracoid (IVPP V16327) in lateral (**A**) and medial (**B**) views. Illustration of proximal end of left humerus in anterior (**C**) and distal (**D**) views. Distal end of right metacarpal I (IVPP V16343) in distal (**E**), extensor (**F**), medial (**G**), flexor (**H**), lateral (**I**) and proximal (**J**) views. Right manual ungual?I (IVPP V16313.a) in medial (**K**), proximal (**L**), lateral (**M**), dorsal (**N**), and ventral (**O**) views. Abbreviations: acr, acromion process; bt, biceps tubercle; corf, coracoid foramen; dpc, deltopectoral crest; for, foramen; ft, flexor tubercle; glen, glenoid; grv, groove; h, head; lcon, lateral condyle; llp, lateral ligament pit; mcon, medial condyle; mcr, medial crest; mlp, medial ligament pit; pdp, proximodorsal process; pvp, posteroventral process; scap, scapula; vg, vascular groove.

A partial scapulocoracoid (IVPP V16327; Fig. 5A,B) consists of the distal scapula and a portion of the coracoid including the glenoid and biceps tubercle. The scapulocoracoid is completely fused and the suture is obliterated. The acromion process is small and projects laterally, but does not have a prominent facet for the furcula. The glenoid faces posteroventrally but is slightly exposed laterally, as is the case in many oviraptorosaurs. The coracoid has a large, knob-like biceps tubercle and is strongly curved posteroventrally. The coracoid foramen is broken (Fig. 5A), but, based on the remaining edge, it was large and positioned directly dorsal to the biceps tubercle.

The humerus is represented solely by the proximal head (IVPP V16340; Fig. 5C,D), which is rounded and projects medially. The deltopectoral crest was apparently small, as in other avimimids, and appears to have been a small tubercle rather than a square, wing-like crest as in caenagnathids and oviraptorids.

A small distal bone end (IVPP V16343) is probably the distal end of the first metacarpal (Fig. 5E–J). The shaft is strongly compressed in the flexor-extensor plane, which matches the broken outline of metacarpal I of MPC-D 100/129. The presumable medial edge of the shaft is attenuated into a sharp crest, which creates a lens shaped cross-section in proximal view. The distal end of the metacarpal is highly unusual and asymmetrical compared to other oviraptorosaurs (Fig. 5E). The medial condyle has a modest ligament pit and is somewhat rounded, but not ginglymoid. The lateral condyle is about half of the size of the medial condyle and there is no articular groove separating them. The lateral condyle is slightly rounded and apparently had a small ligament pit with a posterior flange, although the latter structure is broken.

A small manual ungual (IVPP V16313.a; Fig. 5K–O) is clearly oviraptorosaur based on the relatively large flexor tubercle and a proximodorsal process. Its small size means that it may be referable to Avimimidae, but it is possible that it is from a small caenagnathid or oviraptorid. Unfortunately, much of the proximodorsal process is broken, and the proximal articular surface is worn away. The ungual is curved and the flexor tubercle is large, but not rugose. There is a small posterior groove separating it from the proximal articular surface, which is also the case in some caenagnathids^37,38^. The lateral vascular canal is broad and less well defined than the medial one, and neither bifurcates proximally. The distal end is missing.

**Figure 6:**
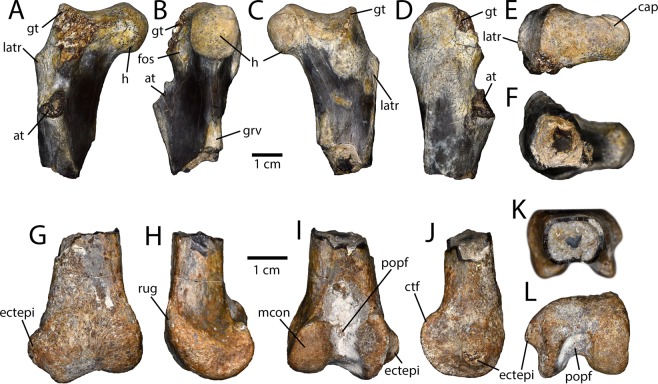
Femora collected from the Iren Dabasu avimimid bonebed. Proximal right femur (IVPP V16334.a) in anterior (**A**), medial (**B**), posterior (**C**), lateral (**D**), proximal (**E**) and distal (**F**) views. Distal right femur (IVPP V16338) in anterior (**G**), medial (**H**), posterior (**I**), lateral (**J**), proximal (**K**), and distal (**L**) views. Abbreviations: at, anterior trochanter; cap, capitate ligament scar; ctf, crista tibiofibularis; ectepi, ectepicondylar tuber; fos, fossa; gt, greater trochanter; h, head; latr, lateral ridge; mcon, medial condyle; popf, popliteal fossa; rug, rugosity.

Two femoral heads (IVPP V16334) were collected, but they differ considerably. One can be identified as avimimid on the basis of a deep cleft between the anterior and greater trochanters (IVPP V16334.a; Fig. 6A–F). In the other specimen (IVPP V16334.b), these structures are fused, which suggests that it may be oviraptorid or, more likely, dromaeosaur. The head of the avimimid femur projects medially and is somewhat spherical, with a distinct lip separating the articular bone from the underlying cortical bone. On the posterior side, there is a notch in the articular bone that extends into a groove on the head for the capitate ligament (Fig. 6E). The head is separated from the greater trochanter by a shallow sulcus, but the entire proximal surface of the femur is formed of spongy epiphyseal bone. This region of articular bone wraps posteroventrally onto the posterior surface of the shaft until a point level with the ventral edge of the head. The greater trochanter is proximally curved and projects as far dorsally as the head. On the anteromedial side of the greater trochanter, there is a deep, pocket-like fossa (Fig. 6B). The dorsal edge of the greater trochanter terminates posteriorly in a rugose mound. On the lateral side of the shaft, level with the anteromedial fossa, there is a prominent tubercle, distal to which the shaft is flat, resulting in a posterior ridge. Only the base of the fingerlike anterior trochanter is preserved. It is separated from the greater trochanter by a wide cleft, as in *Caenagnathasia martinsoni*^39^ and *Microvenator celer*^40^. There are two longitudinal ridges distal to the medial head on the shaft of the femur that form a shallow groove (Fig. 6F). The distal end of a femur (IVPP V16338; Fig. 6G–L) is clearly avimimid, but is unusual compared to *Avimimus nemegtensis* and *A. portentosus*^5^. The bone of the shaft is very thin-walled and rectangular in cross-section (Fig. 6K). The condyles do not flare as widely transversely as other avimimids, but they are similarly robust. The medial condyle is fairly typical for an oviraptorosaur, but has a rugose mound of bone on its anteromedial surface. The lateral condyle has an exceptionally large ectepicondylar tuber, like all avimimids, and is united with the crista tibiofibularis (Fig. 6J), which contrasts other oviraptorosaurs. In contrast to other avimimids, the condyles are separated distally by a deep groove, which is continuous with the deep popliteal fossa.

**Figure 7:**
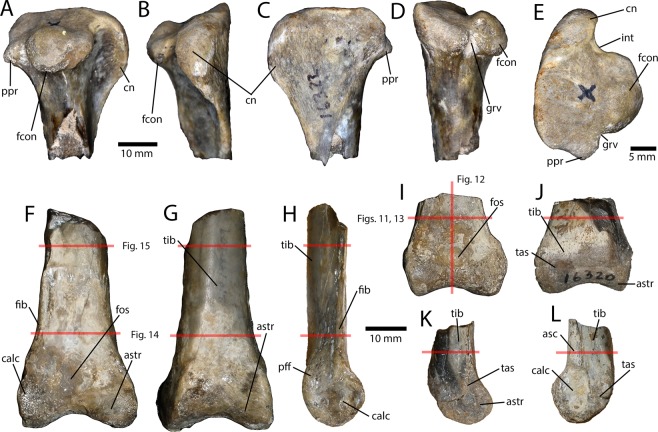
Tibiotarsi recovered from the Iren Dabasu avimimid bonebed. Proximal right tibiotarsus (IVPP V16322.a) in lateral (**A**), anterior (**B**), medial (**C**), posterior (**D**), and proximal (**E**) views. Adult distal right tibiotarsus (IVPP V16337) in anterior (**F**), posterior (**G**), and lateral (**H**) views. Juvenile distal left tibiotarsus (IVPP V16320) in anterior (**I**), posterior (**J**), medial (**K**), and lateral (**L**) views. Red lines in (**F**–**L**) indicate locations of thin sections and corresponding figure numbers. Abbreviations: asc, ascending process of astragalus; astr, astragalus; calc, calcaneum; cn, cnemial crest; fcon, fibular condyle fib, fibula; fos, fossa; grv, groove; int, incisura tibialis; pff, postfibular flange; ppr, posterior process; tas, tibia-astragalus suture; tib, tibia.

Five partial tibiae (Fig. 7) were recovered, three from the proximal end (IVPP V16322.a–c) and two from the distal end (IVPP V16320; IVPP V16337). Two of the proximal ends are from the left, and a larger one is from the right side. Unlike other avimimids^5,21^, the femoral condyle projects posteriorly, which produces a triangular posterior process in medial view (Fig. 7C: ppr). The fibular condyle is large and bulbous (Fig. 7D), more reminiscent of the condition in *Avimimus portentosus*^5^ than the smaller fibular condyle of *Avimimus nemegtensis*^21^. However, the groove separating the fibular condyle posteriorly from the rest of the condyle is much deeper, forming a distinct notch (Fig. 7E). The fibular condyle has two main bulbs, the posterior of which is larger (Fig. 7E). This condyle is larger, more bulbous, and taller dorsally in the larger specimen, which may be related to differences allometric growth. The same is true of the femoral condyle, except instead of becoming more bulbous, it expands medially. The incisura tibialis is semicircular and is relatively smaller in the larger specimen. The cnemial crest is anteroposteriorly small and proximally restricted (Fig. 7C), but robust and transversely thick (Fig. 7B). It has a rounded outline in lateral view, but in the smaller specimens the cnemial crests are slightly squared off ventrally. The cnemial crest is everted laterally and thickens transversely towards its proximal end, where it is bulbous in anterior view. The larger of the two distal ends of the tibiae (IVPP V16337; Fig. 7F–H) is more complete and has fully fused with the astragalocalcaneum. In the smaller specimen (IVPP V16320; Fig. 7I–L), the astragalocalcaneum had begun to fuse distally, but the ascending process is free. Each of the tibiae has a flattened anterior surface which lacks a fibular ridge or groove. The postfibular flange is relatively well developed. In the larger, fused specimen, the fibula is visible fused to the lateral surface of the tibiotarsus (Fig. 7H), as in other avimimids. The calcaneum is laterally concave and it is fused to the astragalus in both specimens, although a suture is visible in the smaller one. The astragalus covers the entire transverse surface of the tibia and has an anterior pit above the distal condyles. There is a small process of the astragalus that overlies the calcaneum in anterior view, as in most other oviraptorosaurs.

**Figure 8:**
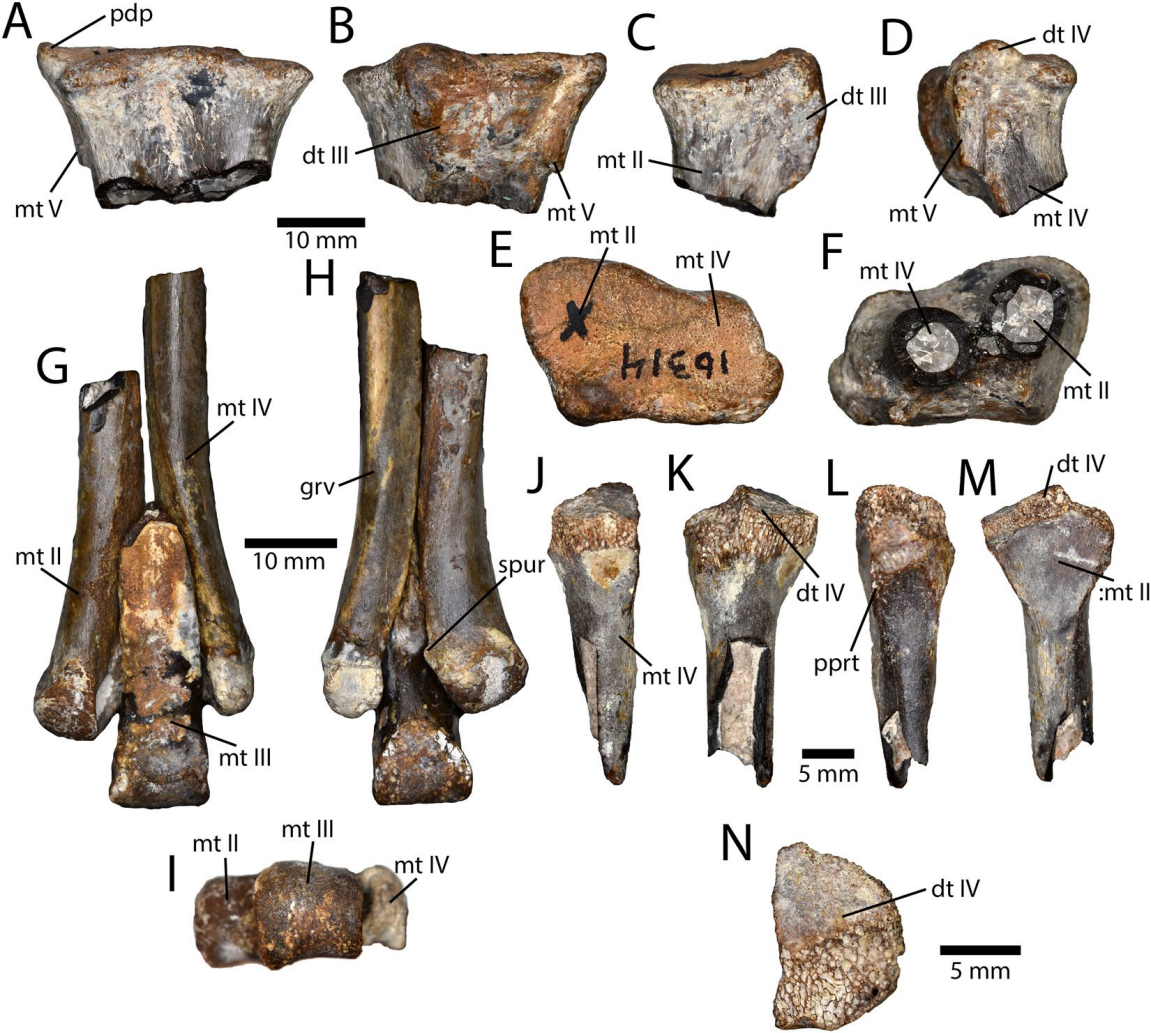
Tarsometatarsi recovered from the Iren Dabasu avimimid bonebed. Proximal right tarsometatarsus (IVPP V16314) in anterior (**A**), posterior (**B**), medial (**C**), lateral (**D**), proximal (**E**), and distal (**F**) views. Rearticulated distal metatarsus (IVPP V16336 and IVPP V16335.b–c) in anterior (**G**), posterior (**H**), and distal (**I**) views. Unfused proximal end of right metatarsal IV (IVPP V16335.a) in anterior (**J**), lateral (**K**), posterior (**L**), medial (**M**), and proximal (**N**) views. Abbreviations: dt III, distal tarsal III; dt IV, distal tarsal IV; grv, groove; mt II, metatarsal II; mt III, metatarsal III; mt IV, metatarsal IV; mt V, metatarsal V. pdp, proximodorsal process; pprt, posterior protuberance; spur, posterolateral spur;:mt II, contact for metatarsal II.

**Figure 9:**
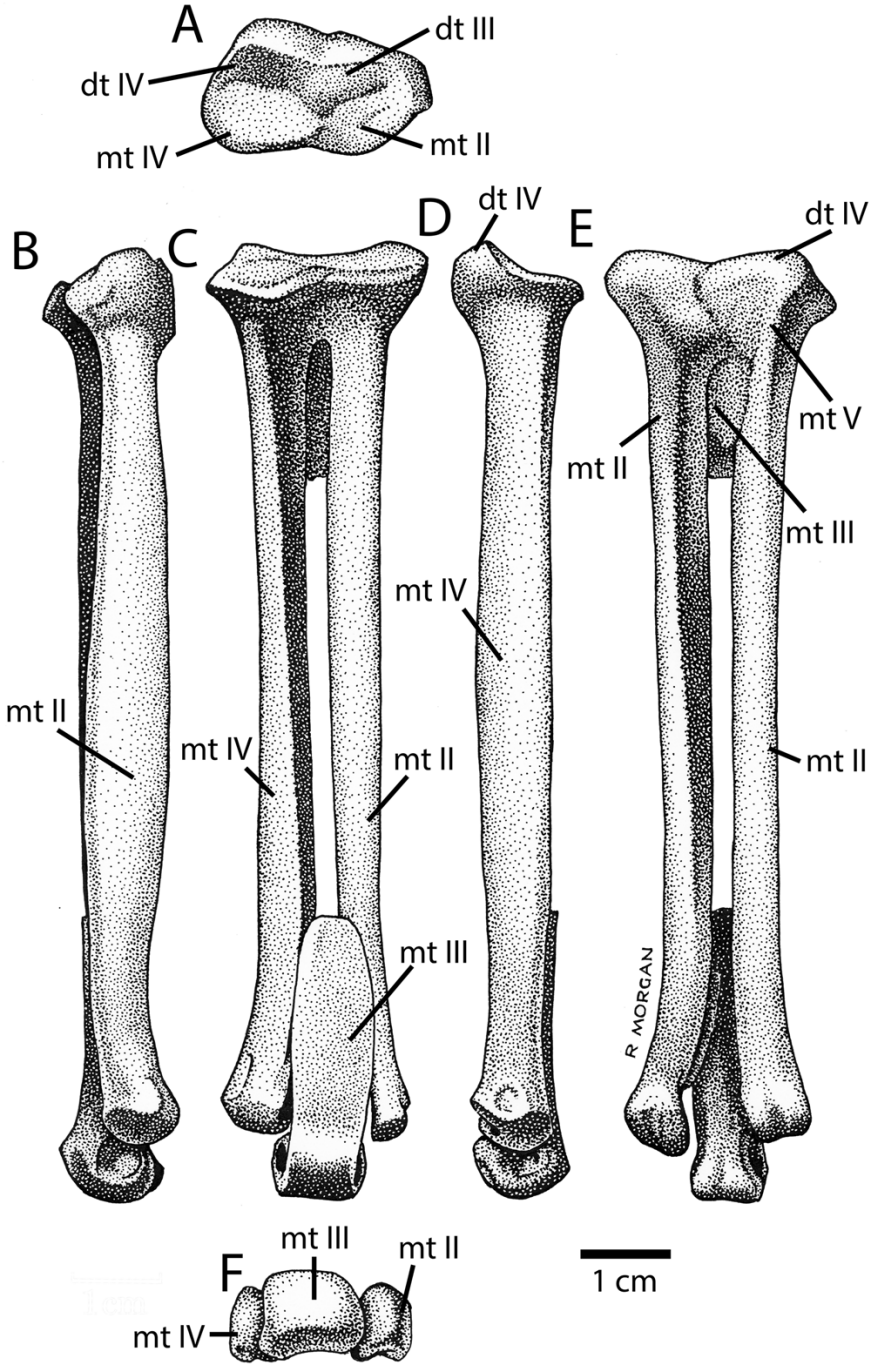
Tarsometatarsus (AMNH 6555) recovered from Erenhot area. Illustration of nearly complete right tarsometatarsus in proximal (**A**), medial (**B**), anterior (**C**), lateral (**D**), proximal (**E**) and distal (**F**) views. Abbreviations: dt III, distal tarsal III; dt IV, distal tarsal IV; mt II, metatarsal II; mt III, metatarsal III; mt IV, metatarsal IV; mt V, metatarsal V.

Numerous partial metatarsals were recovered (Fig. 8), all except one of which (IVPP V16335.a) appear to have been fused proximally. A relatively complete metatarsus (AMNH 6555; Fig. 9) was collected by Peter Kaisen during the AMNH expeditions led by Roy Chapman Andrews in 1923. It is unclear whether this specimen is from the same bonebed assemblage, but it is certainly from the same locality, where the expedition also collected tyrannosaur material. Two size classes are apparent in the metatarsal material. The largest specimens (IVPP V16314 and IVPP V16341) are from the right and left, respectively, and may belong to a single individual. An isolated proximal end of a left metatarsal II (IVPP V16321) is the same size, as is the right tarsometatarsus AMNH 6555. Two left second metatarsals (IVPP V16315 and IVPP V16326) are smaller, especially in the anteroposterior length of the proximal surface of the tarsometatarsus. The fused proximal metatarsus consists of metatarsals II, IV, and V, and distal tarsals III and IV (Fig. 8A–F). Metatarsal III apparently did not contribute to this fused unit, or, if it did, its contribution is minimal. Metatarsal II is larger proximally than metatarsal IV, which appears to increase allometrically. A large bulbous boss on the posterior side of metatarsal II is distal tarsal III, which wraps posteroventrally and is restricted anteriorly (Fig. 8C). This creates a concavity on the proximal articular surface of metatarsal II. Distal tarsal IV is small, but has a prominent proximodorsal process, which may be partly formed by metatarsal V. The shaft of metatarsal V extends ventrally from the proximodorsal process along the lateral edge of the tarsometatarsus (Fig. 8D). Metatarsal IV has a posterior protuberance similar to that formed by distal tarsal III, and together these form a distinct boss, albeit not as large as that of *Elmisaurus rarus*^41,42^. Metatarsal II is slightly longer than metatarsal IV, but more gracile towards its distal end. The shafts of the metatarsals are relatively straight and they do not splay widely at their distal ends. Posteromedial and posterolateral ridges on metatarsal II and IV, respectively, create a posteriorly concave tarsometatarsus, but less so than in *Elmisaurus rarus* because these metatarsals contact each other posterior to metatarsal III. The small, isolated metatarsal IV (IVPP V16335.a; Fig. 8J–N) is fused to distal tarsal IV, but a clean, slightly concave articular surface for metatarsal II (Fig. 8M) indicates that it had not yet begun to coossify with the other metatarsals. Distal to the articular surface, there is a small depression and a rugose patch of bone, which together would have formed a slit for *a. tarsalis plantaris*. There is a lateral ridge on the posterior side of the metatarsal that contacted metatarsal V, which had not yet fused. Medial to this, there is a posterior protuberance (Fig. 8L) as in other oviraptorosaurs with fused tarsometatarsi.

Four isolated distal ends of metatarsals are preserved, representing each metatarsal. A single distal end of metatarsal IV (IVPP V16336) was preserved, and it can be articulated with a left metatarsal III and II (IVPP V16335.b–c; Fig. 8G–I). The distal condyle of metatarsal II is larger than metatarsal IV. It has a deep lateral ligament pit and a small posterior spur that overhangs this pit slightly. The medial ligament pit is small and bordered posteriorly by a ridge. Metatarsal III has a ginglymoid articular condyle with a larger medial side than lateral side (Fig. 8I). The medial ligament pit is deeper, but both are well developed. The shaft is triangular in cross-section and its posterior ridge is rugose distally. Metatarsal IV has a large, rugose facet for metatarsal III and a modest posterolateral ridge. Distal to this ridge, a groove twists from the posterior side to the lateral side as it extends distally. The medial ligament pit is better developed and the condyles are small and transversely narrow.

**Figure 10:**
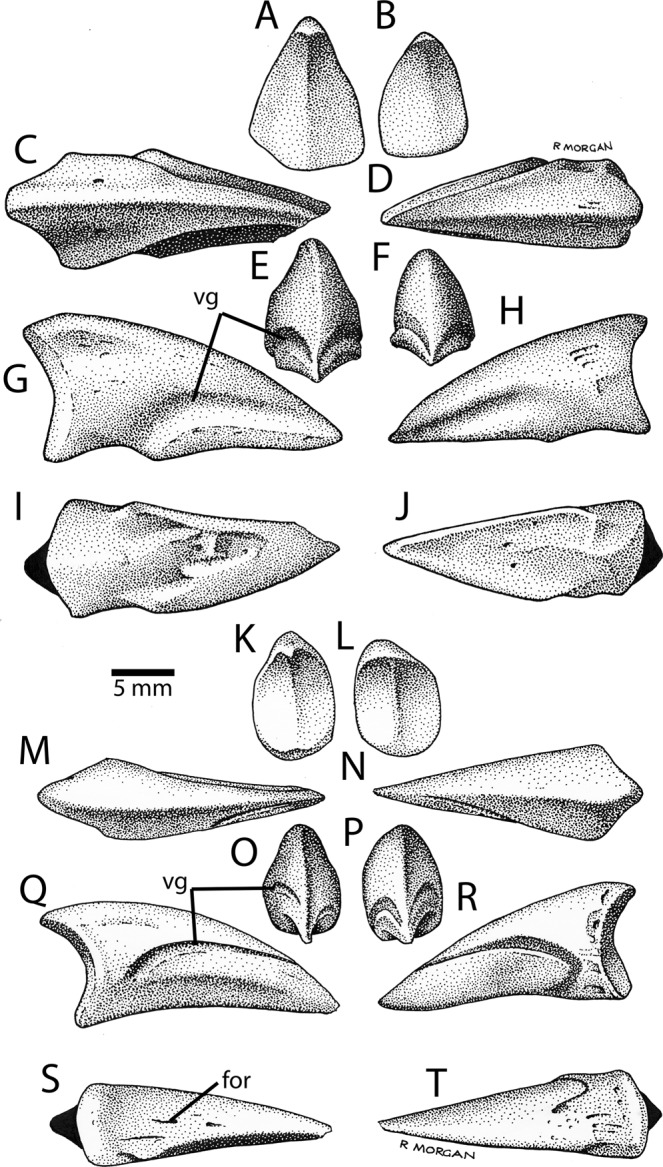
Pedal unguals from the Iren Dabasu avimimid bonebed. Illustrations of digit III morphotype unguals (IVPP V16316.a–b; (**A**–**J**) in proximal (**A**,**B**), dorsal (**C**,**D**), distal (**E**,**F**), lateral or medial (**G**,**H**), and ventral (**I**,**J**) views. Illustrations of digits II/IV morphotype unguals (IVPP V16316.c–d; **K**–**T**) in proximal (**K**,**L**), dorsal (**M**,**N**), distal (**O**,**P**), lateral or medial (**Q**,**R**) and ventral (**S**, om the Iren Dabasu avi) views. Abbreviations: for, foramen; vg, vascular groove.

Thirteen pedal unguals are attributable to an avimimid, but, curiously, no pedal phalanges were recovered. Two morphotypes are represented by the unguals: one is relatively symmetrical and gracile (n = 3; Fig. 10E–H), whereas the other is slanted and asymmetrical (n = 10; Fig. 10A–D). It is likely that the first morphotype corresponds to ungual III-4, whereas the asymmetrical unguals are from digits II or IV. However, whether each ungual represents ungual II-3 or IV-5 cannot be determined and the unguals are virtually identical. Ungual III-4 is gracile and relatively straight, rather than being curved. The proximal articular surface is teardrop shaped and asymmetrical in some specimens. The vascular grooves are low on the unguals and relatively even in height and depth. There is no flexor tubercle, but in its place there is sometimes a slot or foramen that varies in size and depth (Fig. 10F). Ten unguals are the II/IV morphotype. Of these, six slant leftwards in proximal view, and three are angled right. In each case, the direction of inclination corresponds to the side with the more dorsally situated vascular groove, which makes it difficult to tell if these are antimeres or from different digits. Each has a teardrop shaped articulation with a distinct ridge and a prominent posterodorsal process (Fig. 10A,D). The unguals are relatively straight in lateral view and have a series of pits, which probably anchored ligaments, in place of a flexor tubercle (Fig. 10B). The vascular grooves are prominent and the presumable external groove is lower when the tall axis of the claw is oriented vertically. When the ungual is oriented with its ventral surface horizontal, as in life, the vascular grooves become level with each other.

### Osteohistology

**Figure 11:**
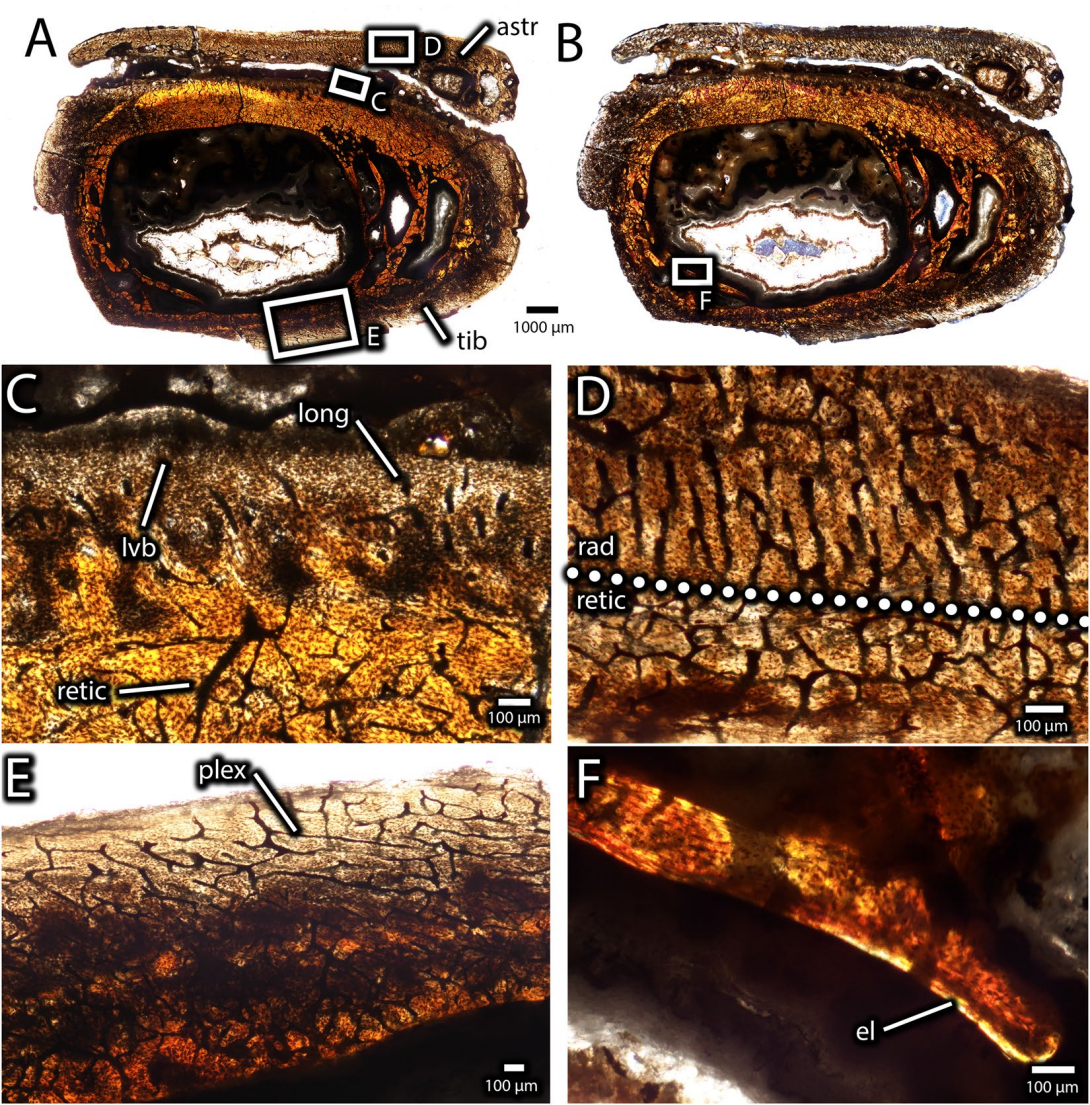
Histology of the distal end of an unfused avimimid tibiotarsus (IVPP V16320). Overview of transverse section under normal light (**A**) and cross-polarized light (**B**), showing tibia, unfused astragalus, and locations of close-up images. Detail (**C**) of anterior edge of tibia under normal light, showing reduced vascularity. Detail (**D**) of zone of radially-oriented vasculature in the astragalus under normal light. Detail (**E**) of posterior surface of tibia under normal light, showing plexiform vasculature. Detail (**F**) of endosteal surface of tibia under cross-polarized light, showing incipient development of endosteal lamellae. Abbreviations: astr, astragalus; el, endosteal lamellae; long, longitudinal vasculature; lvb, low-vascularity bone; plex, plexiform vasculature; rad, radial vasculature; retic, reticular vasculature; tib, tibia.

**Figure 12:**
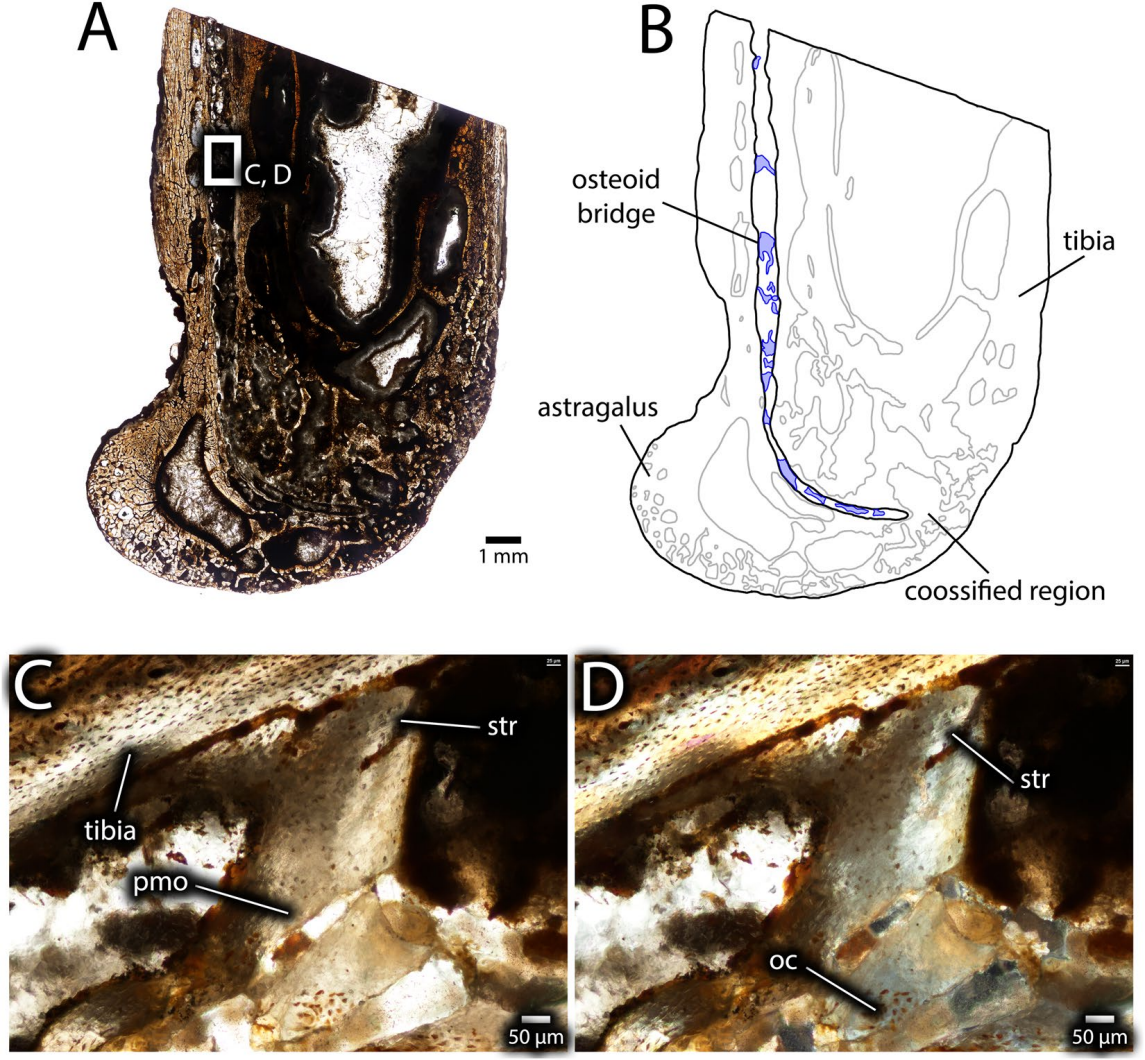
Histology of the distal end of an unfused avimimid tibiotarsus (IVPP V16320). Overview of longitudinal section under normal light (**A**), showing location of close-up images, and interpretive illustration (**B**), showing osteoid bridges (blue). Close-ups (**C**,**D**) of an osteoid bridge under plane-polarized (**C**) and cross-polarized (**D**) light, showing poor clarity of the osteoid suggesting poor mineralization (pmo), clusters of osteocytes (oc), and faint striations in the matrix (str). Abbreviations: oc, osteocyte cluster; pmo, low clarity osteoid; str, striations.

**Figure 13:**
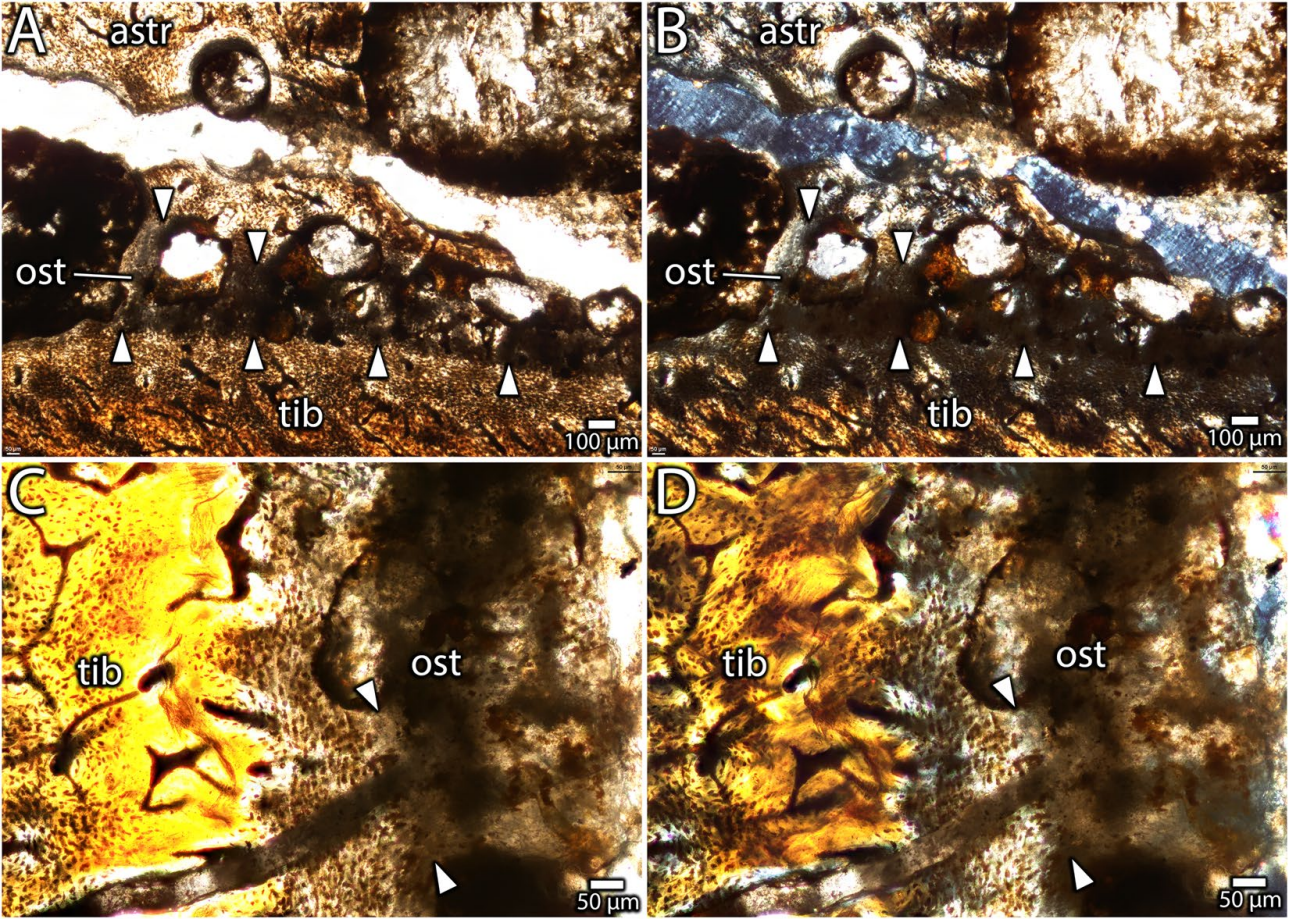
Histological details of astragalus-tibia interface in IVPP V16320 in transverse section. A low clarity osteoid bridge near the lateral edge of the tibia in plane-polarized (**A**) and cross-polarized (**B**) light, showing delineation between the astragalus, tibia and osteoid bridge (arrows). Close-up (**C**,**D**) of transition between the tibia and the osteoid bridge in plane-polarized (**C**) and cross-polarized (**D**) views. Abbreviations: astr, astragalus; ost, osteoid; tib, tibia.

**Figure 14:**
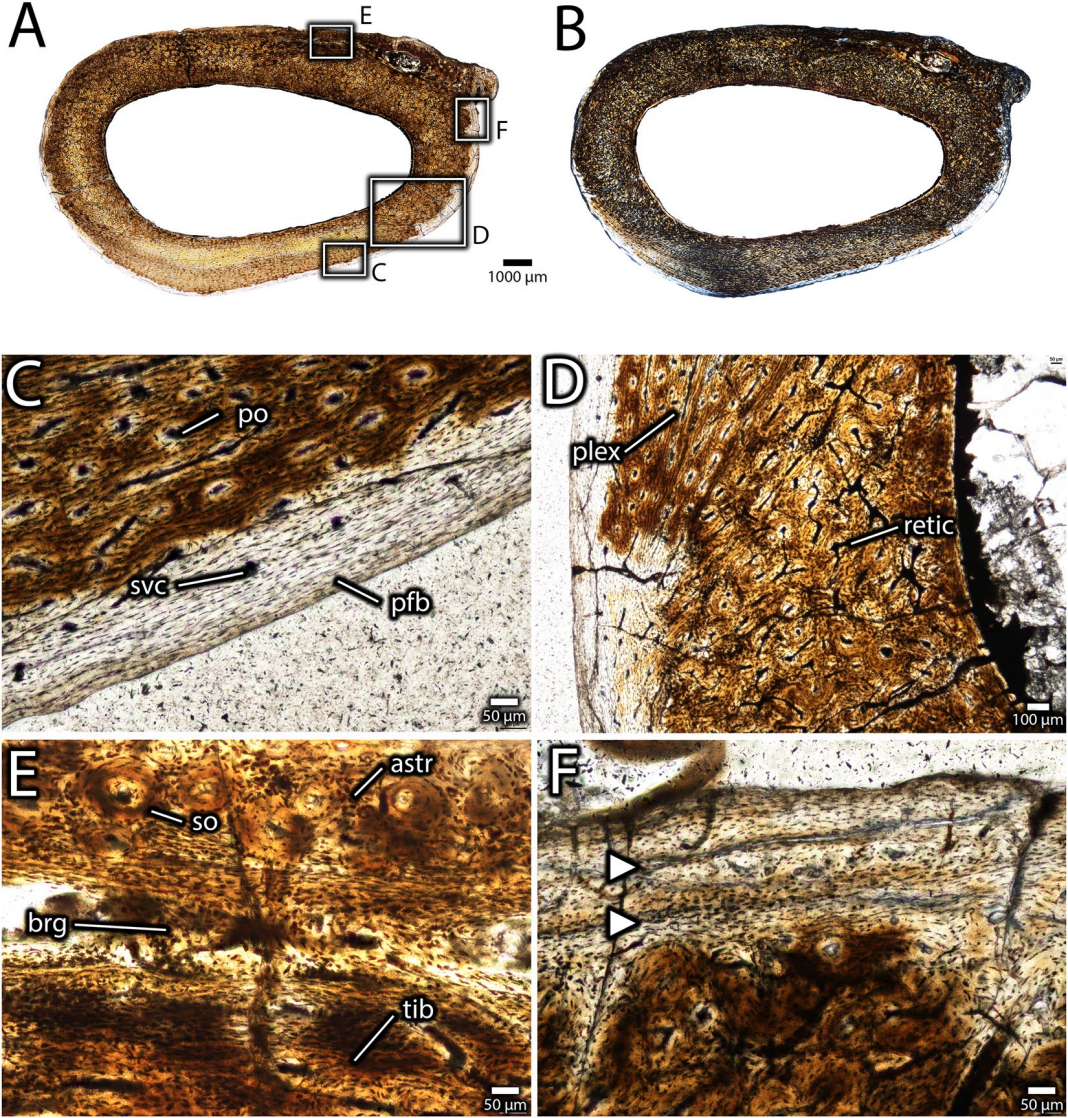
Histology of the distal end of a fused avimimid tibiotarsus (IVPP V16337). Overview of transverse section under normal light (**A**) and cross-polarized light (**B**), showing fusion of tibia, astragalus, and fibula, and locations of close-up images. Detail (**C**) of parallel-fibered bone on the periosteal surface of the tibia with simple vascular canals, indicating slow growth. Detail (**D**) of vascular change in the cortex of the tibia, transitioning from Haversian bone endosteally to primary plexiform bone periosteally along a curved border. Detail (**E**) of interface between the tibia and astragalus, showing fully ossified bridges of intramembranous bone spanning the gap between the bones. Detail (**E**) of growth marks (arrows) in parallel-fibered bone near the periosteal surface on the lateral side of the tibia. Abbreviations: astr, astragalus; brg, intramembranous bridge; pfb, parallel-fibered bone; po, primary osteon; plex, plexiform vascularity; retic, reticular vascularity; so, secondary osteon; svc, simple vascular canal; tib, tibia.

**Figure 15:**
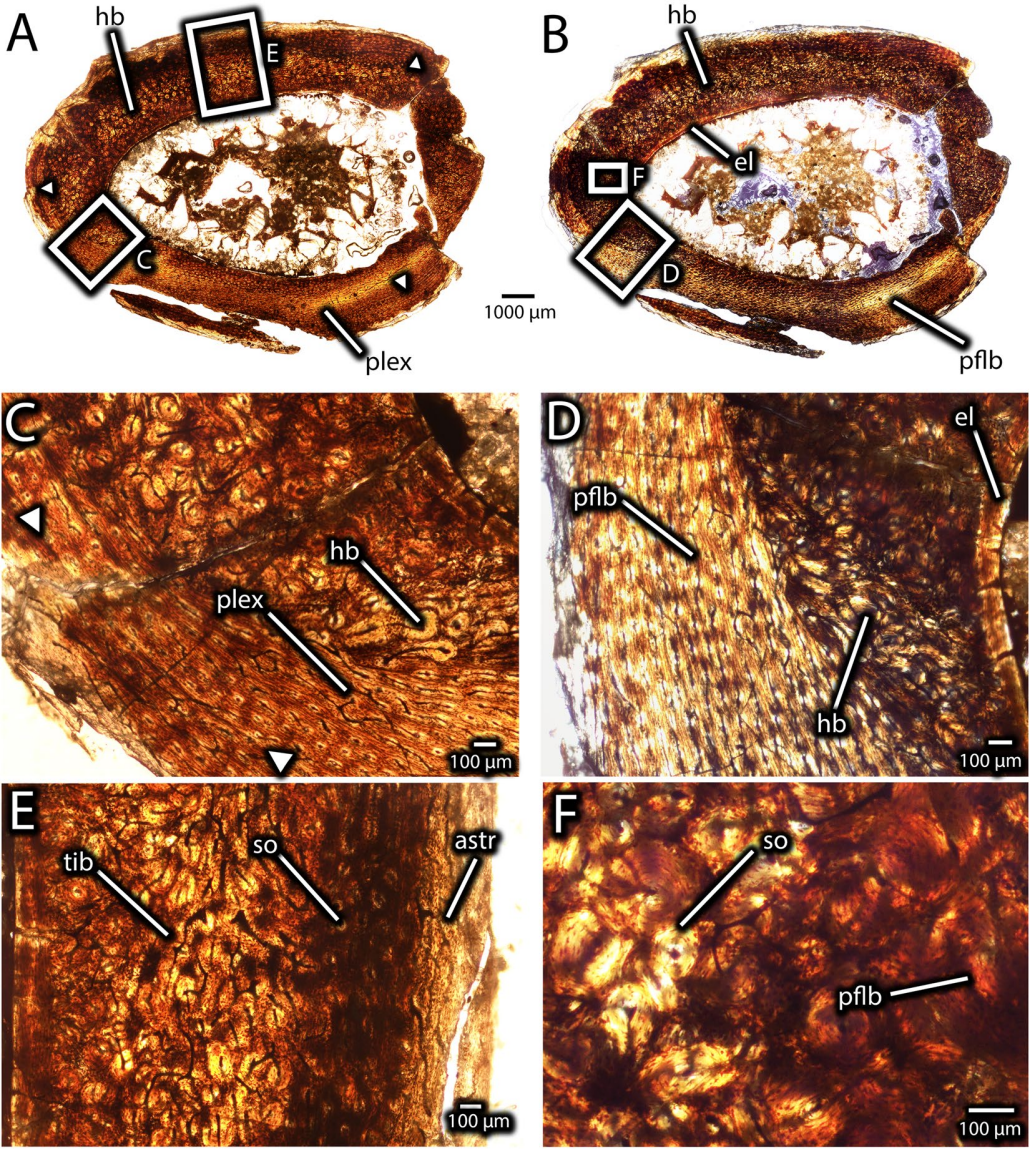
Histology of the midshaft of a fused avimimid tibiotarsus (IVPP V16337). Overview of transverse section under normal light (**A**) and cross-polarized light (**B**) showing posterior zone of plexiform-laminar primary bone and dense secondary remodeling of most of the cortex, as well as locations of close-up images. Detail (**C**) of contact between zone of plexiform-laminar primary bone, and Haversian bone, on the posterolateral side of the tibia. Note growth mark (arrows). Detail (**D**) of transition from endosteal lamellae (far right) to remodeled Haversian bone (center right) to primary fibrolamellar bone with plexiform vasculature (left), under cross-polarized light. Detail (**E**) of tibia-astragalus interface, showing small portion of astragalus (right) and heavily remodeled tibia (left) under normal light. Detail (**F**) of secondary osteons and primary fibrolamellar bone in the inner cortex of the lateral part of the tibia under cross-polarized light. Abbreviations: astr, astragalus; el, endosteal lamellae; hb, Haversian bone; pflb, primary fibrolamellar bone; plex, plexiform vascularity; so, secondary osteon; tib, tibia.

Serial thin sections in transverse (Figs. 11–14) and longitudinal (Fig. 15) planes were made from the two distal tibiotarsi collected from the bonebed (IVPP V16320, IVPP V16337). These specimens vary in the degree of fusion of the tibia and astragalocalcaneum, and therefore may provide some information about the mechanism and timing of fusion. As described previously, the astragalocalcaneum of the smaller specimen (IVPP V16320) is fused distally to the tibia, but the ascending process is not fused. In contrast, these bones are completely and indistinguishably fused in the larger specimen (IVPP V16337), and this fused unit also includes the distal end of the fibula.

#### IVPP V16320

The sections include the tibia and ascending process of the astragalus, but the fibula is not preserved (Fig. 11). The tibia of this specimen is composed entirely of primary fibrolamellar bone, but the texture and vascularization vary considerably throughout the cortex when viewed in transverse sections (Fig. 11). The medullary cavity is large and spanned by several trabeculae of primary fibrolamellar bone, rather than endosteal lamellae. The endosteal surface is unfinished in most areas and scalloped Howship’s lacunae indicate it was being actively resorbed in these areas. However, some parts of the endosteal margin, especially towards the lateral and posteromedial portions of the bone, have thin endosteal lamellae indicating secondary deposition (Fig. 11F). The vascularity of the cortex is highly disorganized, consisting of web-like, predominantly reticular vascular canals. Two exceptions are the anterior and posterior surfaces of the tibia, where vascular canals are more organized. On the anterior surface of the tibia (Fig. 11E), vascularization is reduced and in some areas, a greater proportion of canals are oriented longitudinally, although vascularity is reticular overall. In the posterior half of the tibia, vascular canals become slightly more organized towards the periosteal surface and approach a sub-plexiform orientation (Fig. 11E). Osteons are moderately well developed and osteocyte lacunar density^43^ (~53,000/mm^3^) is relatively consistent throughout the cortex.

The ascending process of the astragalus is represented in transverse sections by a thin ribbon of bone that is separated from the tibia by a gap of approximately 100 µm (Fig. 11A,B). Like the tibia, it is composed entirely of primary fibrolamellar bone, but it differs considerably in vascular orientation. Towards the anterior and posterior edges of the astragalus, vasculature is reticular in orientation, but there is a narrow band in the middle of the bone where vasculature is oriented mostly radially (Fig. 11D). Three large vascular canals pierce the lateral part of the ascending process in the transverse plane. The largest evidently separated the tibia and astragalus, and it is lined by endosteal lamellae.

Whereas the astragalus and tibia appear mostly separate in transverse sections, the extent of fusion between these bones is more evident in the longitudinal plane (Fig. 12). The posterodistal extreme of the contact is completely ossified, but otherwise a narrow channel separates the astragalus from the anterior and distal surfaces of the tibia (Fig. 12B). The interface between the astragalus and tibia provides information about the mechanism of fusion between these bones. In both longitudinal and transverse views, several bridges of tissue with reduced clarity, which is likely due to its originally low mineralization level, span this gap (Figs. 12 and 13). This tissue has fewer osteocyte lacunae than the surrounding bony tissue, and these osteocytes tend to be clumped rather than evenly distributed. In longitudinal view, this tissue has striations that resemble Sharpey’s fibers (Fig. 12C,D), although they are less distinct. Together, these features suggest that the tissue is poorly mineralized osteoid, characterized by a high proportion of organic material with low optical clarity. In this case, each clump of osteocyte lacunae may represent ossification centres, loci from which mineralization of the osteoid spreads^44^. Striations within the osteoid may represent partly mineralized collagen fibers. The osteoid is continuous with the periosteal surfaces of both the tibia and the astragalus and these surfaces show a gradient of clarity suggesting that the osteoid was being deposited by the periostea of both bones (Fig. 13A,B). This is also supported by the greater clarity of the osteoid adjacent to the periosteal surfaces, which suggests it was more mineralized, and was therefore deposited before the osteoid with lower clarity in the middle of the interface. However, under cross-polarized light (Fig. 13B,D) the extinction pattern of the osteoid differs from the more typical extinction patterns of fibrolamellar bone in both the astragalus and the tibia, indicating that it was deposited separately from the normal growth of these elements. No chondrocytes or calcified cartilage can be detected at the interface of the tibia and astragalus, suggesting that the osteoid in this region was deposited through intramembranous ossification or metaplasia rather than endochondral ossification.

#### IVPP V16337

Because more of IVPP V16337 was preserved, sections were made from both the distal end (Fig. 14), to elucidate the process of tibiotarsal fusion, and from the proximal end (Fig. 15). The latter is closer to the midshaft of the bone and therefore more useful for skeletochronology.

The distal thin sections (Fig. 14) show the tibia, astragalus, and fibula, all of which are fused into a single unit. Although the cortex is still predominantly composed of primary fibrolamellar bone, there is significantly more secondary remodelling than IVPP V16320. Osteocyte lacunae are relatively dense (~45,000/mm^3^) throughout the cortex, but they are patchily distributed, because they are denser in the primary bone than the secondary osteons. Unlike IVPP V16320, the entire medullary cavity is surrounded by endosteal lamellae, and these lamellae are thicker anteriorly than posteriorly. Most of the anterior portion of the tibial cortex is secondarily remodeled, but where primary bone remains, it has reticular vascularity similar to IVPP V16320. A thin band of primary, low vascularity, parallel-fibered bone is present at the periosteal surface on the lateral, medial, and posterior sides of the tibia (Fig. 14C). Endosteal to the parallel-fibered bone, the cortex of the posterior side of the tibia differs in vascular arrangement from the rest of the cortex. This region is still primary fibrolamellar bone, but it has plexiform-laminar vascularity, rather than reticular vascularity, and variable thickness towards the lateral and medial sides of the cortex, where it tapers out (Fig. 14D). The variable thickness of this zone around the cortex suggests a considerable degree of cortical drift, as does the absence of secondary remodelling in this region.

The contact between the astragalus and tibia is extensively remodelled (Fig. 14E), and some of these areas are composed entirely of Haversian bone. In other areas, however, the intervening space between the secondary osteons is formed by either primary osteonal bone, or, more towards the astragalus, by parallel-fibered bone. Indeed, much of the primary bone of the astragalus is nearly avascular parallel-fibered bone (Fig. 14E). There is a notable transition in texture from the reticular fibrolamellar bone of the tibia to the less vascularized parallel-fibered bone of the astragalus. However, in most areas these two zones are abutting, rather than separated by a transitional tissue as in the smaller specimen (IVPP V16320). Towards the lateral side of the tibiotarsus, however, a few small gaps between the bones remain. Spanning these gaps are bridges of fully ossified tissue that probably correspond to the osteoid bridges visible in IVPP V16320, but with more complete mineralization (Fig. 14E). Like in IVPP V16320, a large vascular canal separates the tibia and astragalus towards the lateral edges of each bone. It is likely that this is the same structure as in the former specimen, and it probably conducted nerves and vasculature towards the distal end of the tibiotarsus. No growth marks are visible in the anterior part of the cortex, although they could possibly have been obscured by secondary remodelling. However, there are two growth marks within the periosteal zone of parallel-fibered bone, which are better defined on the medial and lateral sides of the tibia (Fig. 14F). The parallel-fibered bone near the periosteal surface of the tibia is poorly vascularized, consisting mainly of simple vascular canals rather than well developed osteons, indicating slow growth (Fig. 14C).

The more proximal section (Fig. 15) preserves mostly the tibia, and only small fragments of the fibula and ascending process of the astragalus are visible. Like in the more distal section, there is significantly more remodelling of the cortex than in the smaller specimen (IVPP V16320), and most of the tibia is composed of Haversian bone (Fig. 15A,B), as in the more distal section. Like the more distal section, this secondary remodelling is concentrated on the anterior portion of the tibia. The endosteal lamellae are well-formed and thick on the anterior part of the medullary cavity. As in the more distal sections, the posterior part of the tibia is formed of primary fibrolamellar bone with plexiform-laminar vascularity. However, in contrast to further distally, this bone type encircles more of the periosteal surface, extending anteriorly where the fibula and astragalus taper. Along the lateral and presumably medial surface (the latter is broken), this region of bone is much narrower and most of the cortex is formed by Haversian bone. One growth mark is visible in the posterior cortex, corresponding in position to the more endosteal growth mark in the distal sections (Fig. 15C). A second growth mark may be preserved near the periosteal surface, but the surface is too damaged to trace it around the entirety of the bone. Regardless, the bone at the periosteal surface is parallel-fibered and less vascularized, indicating a low rate of growth and possibly incipient development of an external fundamental system.

## Discussion

The material from the Iren Dabasu Formation is clearly referable to Avimimidae on the basis of several features. For example, the morphology of the cervical vertebrae is identical to both *Avimimus portentosus* and *Avimimus nemegtensis*, and their combination with dorsal vertebrae lacking pleurocoels is a hallmark character of avimimids. The caudal vertebrae are similarly distinctive because they lack the elongation of the centra present in other coelurosaur groups, but also lack the pleurocoels that are present in more derived oviraptorosaurs like caenagnathids and oviraptorids. Furthermore, the fusion of the tibiotarsus and tarsometatarsus are distinctive and differ morphologically from these compound elements in other Late Cretaceous theropods like alvarezsaurs^45^, birds^46^, and caenagnathids^42,47^. However, the Iren Dabasu Formation material also shows some differences from both *Avimimus portentosus*^5,17^ and *Avimimus nemegtensis*^18,20,21^. For example, the cervicodorsal vertebrae differ in number (three with hypapophyses) from those of *Avimimus portentosus* (MPC-D 100/129^6^), although cervicodorsal number in *Avimimus nemegtensis* is unknown. The distal condyles of the femur (Fig. 6) are separated much more deeply than is typical in avimimids, and metatarsals II and IV (Fig. 8) are much more disparate in size. Unfortunately, the available material from the Iren Dabasu bonebed is too incomplete to confidently erect a new taxon, but future preparation of the Russian material (or collection of new material) may result in its taxonomic distinction from other avimimids.

The tibiotarsi from Iren Dabasu provide information about the growth of avimimids and the process of tibiotarsal fusion. Despite the onset of fusion of the tibia and astragalocalcaneum in IVPP V16320, numerous lines of evidence suggest it was a young, rapidly growing animal. The predominance of reticular vasculature indicates that this individual was growing quickly, similar to other young oviraptorosaurs^48^. The absence of growth marks suggests that the animal was less than one year old at the time of death, especially considering that secondary remodelling, which could have obscured growth marks, is limited (Fig. 11). However, the endosteal margin was clearly subjected to resorption, as shown by the scalloped Howship’s lacunae and the deposition of endosteal lamellae in some areas (Fig. 11F). Therefore, it is conceivable that one or more growth marks has been removed by expansion of the medullary cavity and thus, this individual is best considered as a rapidly-growing juvenile of indeterminate age. Regardless, this indicates that fusion of the tibiotarsus began earlier in ontogeny than expected, during a period of rapid growth rather than after growth had slowed. The combination of rapid growth and fusion of IVPP V16320 is surprising, considering that fused avimimid tibiotarsi in bonebeds vary by less than 10% in length, and fusion has been suggested as a sign of skeletal maturity^20^.

The presence of poorly mineralized osteoid at the interface of the astragalus and tibia in IVPP V16320 provides insight on the mechanism of fusion between these two bones. The periostea of both the astragalus and the tibia contribute to this new ossification (Fig. 13), which proceeds initially by the establishment of several bridges (Fig. 12) followed by infill of the remaining spaces (Fig. 14). The absence of calcified cartilage or chondrocytes argues against endochondral ossification as a mechanism for fusion. Instead, the poorly mineralized osteoid bridges could be evidence for fusion by primary intramembranous ossification, similar to symphyseal closure in some mammals^49,50^, or metaplasia, via mineralization of an intervening soft tissue^51^. Which of these mechanisms is more likely is unclear. However, some signs may point more towards an intramembranous origin than a metaplastic origin. In particular, osteocyte lacunae—identified by the presence of canaliculi—are apparent in the osteoid matrix (Fig. 12C,D) and the fully ossified tissue (Fig. 14E), and these would not be expected of metaplastic tissue^51^. These tissues also lack the herringbone pattern characteristic of metaplastic bone^51^, although in some areas they do appear fibrous (Fig. 12C,D). However, intramembranous bone is usually high in porosity and vascularity, which is not the case in the fusion tissue observed here. A broader histological sample covering more of the fusion interval could provide evidence to distinguish these two options.

**Figure 16:**
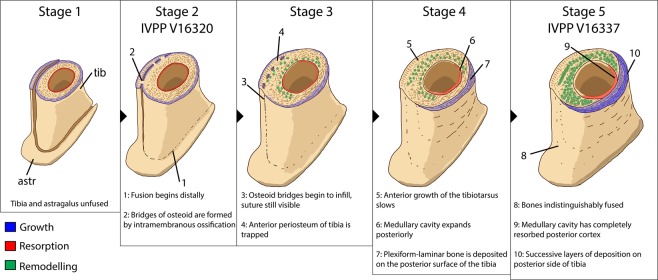
Hypothesized sequence of events during the growth and fusion of the tibiotarsus in avimimids. Each illustration shows the tibiotarsus in oblique dorso-postero-medial view, transversely sectioned at the same plane as Figs. 11 and 14. Fusion begins distally by the formation of osteoid bridges via intramembranous ossification, and proceeds proximally, entrapping the periosteum of the anterior part of the tibia. Cortical drift results from deposition of fibrolamellar bone on the posterior part of the tibia and more slowly growing parallel-fibered bone elsewhere. Blue indicates areas of bone deposition, red indicates areas of bone resorption, and green indicates secondary remodelling. Images not to scale.

IVPP V16337 (Figs. 13 and 14) reveals aspects of growth after fusion of the tibiotarsus is complete. Once the gap between the astragalus and tibia was filled, the interface of the bones remodelled (Fig. 14E). Changes occurred in cortical growth as well. The absence of the parallel-fibered and the plexiform-laminar zones in the cortex of IVPP V16320 (Fig. 11) suggests that these regions in IVPP V16337 were deposited after fusion of the tibiotarsus was complete. The change in growth style, the variable thickness of these zones around the cortex, and the strongly asymmetrical secondary remodelling pattern suggest cortical drift, possibly related to fusion of the tibiotarsus (Fig. 16). These signals suggest that the tibiotarsus grew more slowly anteriorly than posteriorly, although why this occurred is unclear. The posterior zone of primary plexiform-laminar bone suggests that more periosteal primary bone was deposited on the posterior part of the tibiotarsus than the anterior part (Figs. 14D and 15C,D), and this is supported by the greater prevalence of secondary remodelling in the anterior portion of the cortex. This indicates that cortical thickness was maintained by posterior expansion of the medullary cavity, which also resulted in thicker endosteal lamellae anteriorly than posteriorly (Fig. 16). Tibiotarsal fusion may also explain the reduced thickness of this zone of new growth on the medial and lateral sides of the tibiotarsus (Fig. 15D). Coossification of the tibiotarsus could have restricted the maximum transverse dimensions of this element by fixing the proportions of the ankle joint. Accordingly, less bone was deposited on the medial and lateral sides of the tibia, and the posterior surface became the primary area for cortical expansion. Because at least two growth marks are preserved in the zone of parallel-fibered bone (Fig. 14F), this individual was at least two years older than IVPP V16320. The zone of parallel-fibered bone with simple vascular canals at the periosteal surface suggests that this individual was growing slowly and had approached maximum body size. It is therefore best regarded as a skeletally mature individual older than two years, confirming that growth was limited after fusion of the tibiotarsus.

Together, these results elucidate the growth patterns of avimimids. Growth was initially rapid and juveniles approached maximum body size rapidly, possibly within a single year. At this point, fusion of the tibiotarsus occurred via intramembranous or metaplastic ossification, presumably in conjunction with fusion elsewhere in the skeleton^17,20^. After fusion, the tibiotarsus grew more slowly anteriorly than posteriorly, resulting in cortical drift (Fig. 13B). Body mass may still have increased after tibiotarsal fusion, accommodated for by anteroposterior expansion of the tibiotarsus, but it is unlikely that the linear dimensions of the hindlimb or animal increased appreciably. This appears to be evident in the material collected from the *Avimimus nemegtensis* bonebed at Nemegt, where fused tibiotarsi vary in length by less than 10%^20^. This indicates determinate growth like in birds, mammals, and presumably many other dinosaurs^52^. This growth style contrasts that of most mammals and dinosaurs^53,54^, however, because avimimids did not decrease growth rate gradually. Instead, histological evidence indicates that young avimimids were in the most rapid phase of their growth when the tibiotarsus fused and growth rate reduced only after this fusion was completed. If the sample at the Nemegt Bonebed is representative of avimimids more broadly, individuals would have grown to about 90% of maximum body size before growth rate declined. This growth pattern contrasts the expectation that avimimids remained small because of lower growth rates, like other small non-avian theropods^55^. Whether sexual maturity coincided with skeletal fusion is unclear, but it is unlikely that it preceded fusion, based on the evidence for rapid growth in IVPP V16320. Typically, sexual maturity is accompanied by a stark decrease in growth rate^56–59^, resulting in a sigmoidal growth curve for most vertebrates, but IVPP V16320 shows no signs of growth rate decrease.

A survey of cranial suture microstructure in archosaurs showed that sutures in extant emu (*Dromaius novaehollandiae*) and mammals typically close by means of periosteal ossification, but that extinct archosaurs had greater diversity in suture closure more reliant on metaplasia^50^. That study commented that sutures in extant avians were more similar to those of mammals than other archosaurs because intramembranous ossification was the main mode of suture closure, but that it was likely this arose at some point in non-avian saurischians, which were not sampled. If this avimimid suture closure is intramembranous in nature (as discussed previously), it may signify that this avian characteristic had already evolved in Pennaraptora. However, Bailleul and Horner^50^ also note that rapid maturation in birds may have limited the breadth of tissues that could develop. Accordingly, fusion by intramembranous ossification in avimimids—if present—may be the result of convergence on an accelerated growth style. Establishing the origin of this type of suture closure within Saurischia will require a broader sample.

The lack of *in situ* material collected from the site precludes detailed taphonomic analysis, but some information on the taphonomy can be gleaned. The pristine condition of most material, especially considering that it had been exhumed for 30 years prior to collection, suggests that the bonebed material was buried relatively rapidly^60^. However, the disarticulation of the bones, except for fused compound elements (tibiotarsi, tarsometatarsi), indicates that they were exposed long enough for the flesh to decay. Representation of a variety of elements from the entire skeleton argues against hydraulic concentration of dissociated material and suggests that the individuals perished together in a mass mortality^61^. Accordingly, the bonebed assemblage supports previous suggestions of gregarious behaviour in avimimids^20^. However, some material from the bonebed pertains to other taxa, including a dromaeosaur and an indeterminate ornithischian, and it is therefore possible that the assemblage was reworked after initial deposition.

The histological results presented here raise doubts regarding the assertion of Funston *et al*.^20^ that the specimens in the Nemegt *Avimimus* bonebed are all adults or subadults. In particular, the onset of tibiotarsal fusion in IVPP V16320, despite histological immaturity, suggests that fusion of compound bones in avimimids begins early in ontogeny, possibly within the first year of life. In any case, the tibiotarsus began to fuse before growth rate decreased, and its fusion is therefore not necessarily an indicator of advanced age, but it may effectively indicate proximity to skeletal maturity. The absence of fusion in specimens at the *Avimimus nemegtensis* bonebed, therefore, probably indicates that they are young juveniles, rather than subadults, regardless of their size. This would resolve the unusual rarity of juvenile individuals in that bonebed and would suggest that a foraging assemblage is a more likely explanation for the assemblage than ontogenetically-segregated lekking behaviour, which was considered a possibility by Funston *et al*.^20^. However, it is possible that the onset of fusion was ontogenetically or taxonomically variable in avimimids and verification of ontogenetic stage in the *Avimimus nemegtensis* bonebed individuals will require future histological analysis of those specimens. Regardless, the presence of both juveniles (IVPP V16320) and adults (IVPP V16337) in the Iren Dabasu bonebed assemblage indicates that at least some avimimids grouped in mixed-age flocks, which is unusual in theropods^20,62^. Typically, non-avian theropod groups were formed mostly of juveniles^62,63^, although some bonebeds have more even representation of the population^64,65^. It is possible that mixed-age flocks were more common in avimimids because rapid growth trajectories resulted in minimal differences in body sizes of juveniles and adults. This possibility is supported by the similarity in size of IVPP V16320 and IVPP V16337, despite the much earlier ontogenetic stage of the former. This is also the case in the larger sample from the *Avimimus nemegtensis* bonebed, where even the smallest unfused bones are more than 80% the length of the largest adult elements^20^. The proximity in body size and functional similarity of juvenile and adult avimimids contrasts with other non-avian dinosaurs, where assemblages are comprised either of functionally-similar, age-segregated groups^62,66–70^, or mixed-age groups of different body sizes and, presumably, ecology^64,65,71^. Evidence from other oviraptorosaurs suggests that avimimids were predominantly herbivorous^21,72–74^ and thus diet may not have varied as much through ontogeny as in more predatory theropods^75,76^. As ontogenetic niche shift was likely responsible to some degree for ontogenetic segregation in these other dinosaur groups, its absence may have facilitated mixed-age foraging behaviour in avimimids. The same may have been true for other small, fast-growing theropods.

## Materials and Methods

Material examined (AMNH 6555; IVPP V16313–14, V16316–19, V16321–45) consists of disarticulated float from spoil piles of the Sino-Soviet excavation. The specimens represent all regions of the skeleton (Fig. 2), although elements are incomplete.

Histological thin-sections of two distal tibiotarsi (IVPP V16320 and V16337) were made by vacuum-embedding the specimens in Buehler Epothin Resin or Castolite AC polyester resin and cutting the billet using a Hillquist Thin Section Machine or an Isomet 1000 Precision Sectioning Saw. Billets were adhered to plexiglass slides using Buehler Epothin Resin or 3M Cyanoacrylate glue. Thin sections were ground and polished from the mounted slides using a variety of grits on a lapidary wheel or by hand on a glass plate. Osteocyte lacunar density was calculated from Z-stacked images using the method of Cullen *et al*.^43^ Osteohistological terminology follows Padian and Lamm^52^.

## Data Availability

All data used in the study is presented in the manuscript and available upon request from the corresponding author.
